# The Power of CRISPR-Cas9-Induced Genome Editing to Speed Up Plant Breeding

**DOI:** 10.1155/2016/5078796

**Published:** 2016-12-20

**Authors:** Hieu X. Cao, Wenqin Wang, Hien T. T. Le, Giang T. H. Vu

**Affiliations:** ^1^Leibniz Institute of Plant Genetics and Crop Plant Research (IPK), Corrensstrasse 3, Gatersleben, 06466 Stadt Seeland, Germany; ^2^School of Agriculture and Biology, Shanghai Jiaotong University, 800 Dong Chuan Road, Shanghai 200240, China; ^3^Institute of Genome Research, Vietnam Academy of Science and Technology, 18 Hoang Quoc Viet Road, Cau Giay, Hanoi, Vietnam

## Abstract

Genome editing with engineered nucleases enabling site-directed sequence modifications bears a great potential for advanced plant breeding and crop protection. Remarkably, the RNA-guided endonuclease technology (RGEN) based on the clustered regularly interspaced short palindromic repeats (CRISPR) and CRISPR-associated protein 9 (Cas9) is an extremely powerful and easy tool that revolutionizes both basic research and plant breeding. Here, we review the major technical advances and recent applications of the CRISPR-Cas9 system for manipulation of model and crop plant genomes. We also discuss the future prospects of this technology in molecular plant breeding.

## 1. Introduction

Under pressure of rapid population growth, climate change, and agricultural pests and diseases, the next green evolution with new technologies is required to address and provide novel genetic variations to improve yield, quality, and resistance against biotic and abiotic stresses in crop plants. During the last two decades several crop genomes have been altered by introduction of one or more foreign genes of high agronomic values to overcome the limitations of conventional breeding techniques. Promises as well as critics on such genetically modified crops have been discussed intensively in other reviews (e.g., [1–3]) and are beyond the scope of this paper. Alternatively and more powerful, genome editing allows precise and predictable changes to be made to the crop genetic materials and currently revolutionizes crop breeding (e.g., [4–7]).

**Figure 1:**
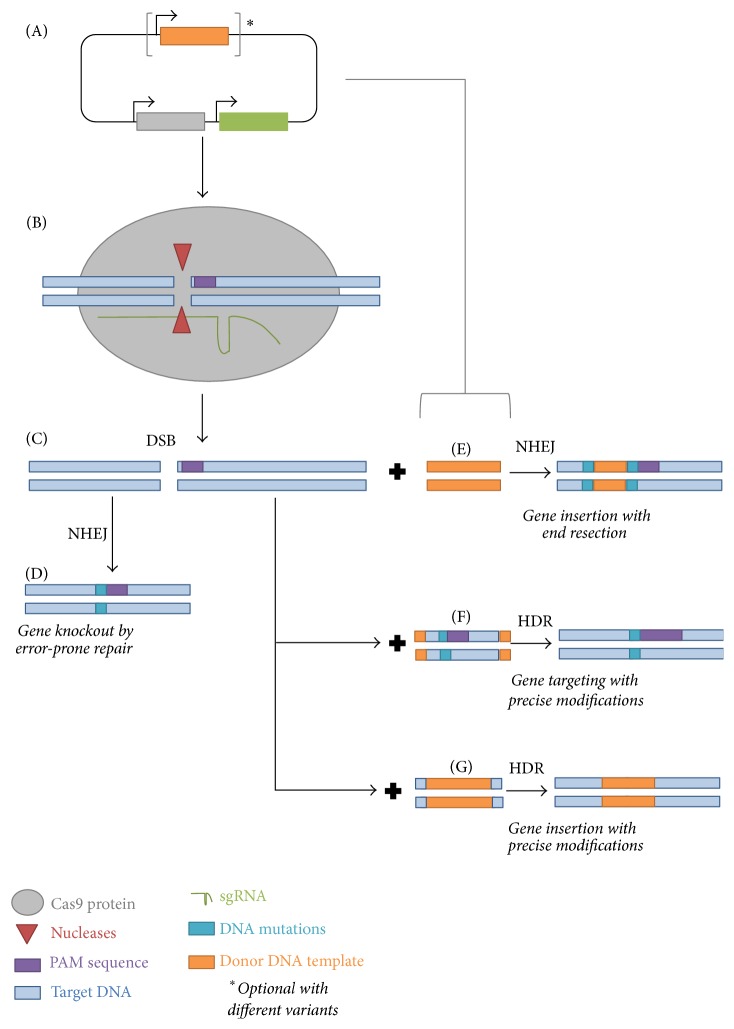
Overview of CRIPR-Cas9 technology for plant genome editing. (A) The most widely used engineered CRISPR-Cas9 system in plants utilizes a plant-codon-optimized Cas9 protein and (could be more than one) single-guide RNA (sgRNA). Optionally, the gene targeting system with geminivirus replicons includes an additional donor DNA template. (B) In plant cells, sgRNA associated with Cas9 nuclease mediates cleavage of target DNA sites that are complementary to the sgRNA and locate next to a PAM sequence. (C) Cas9-induced double-strand DNA breaks (DSBs) can be repaired by nonhomologous end-joining (NHEJ) or homology-directed repair (HDR) pathways. (D) Imprecise NHEJ-mediated repair can generate insertion and/or deletion mutations with variable length at the site of the DSB. These InDels can cause out-of-frame mutations in the coding sequences of the target genes, resulting in gene knockout. (E) In the presence of donor DNA, NHEJ can insert the donor DNA into the site of the DSB together with possibly additional InDel mutations. HR-driven repair can produce precise modifications, including point mutations (F) or insertions from double-/single-strand DNAs as donor templates (G).

Genome editing with site-specific nucleases introduces DNA double-strand breaks (DSBs) at a target site, stimulating cellular DNA repair mechanisms and subsequently resulting in various types of genome modifications such as targeted mutagenesis, gene insertion, or gene replacement. The two main DSB repair pathways in eukaryotic cells are nonhomologous end-joining (NHEJ) and homologous recombination (HR). NHEJ often can cause insertions or deletions, potentially producing a gene knockout. When repair templates (regions of homology to the sequence surrounding the DSB) are available, the HR machinery can be recruited to achieve precise modifications (homology-directed repair, HDR), such as gene replacement or gene insertion (Figure 1). Apparently, NHEJ is the most commonly employed DSB repair mechanism in many organisms, including higher plants [8, 9].

The latest ground-breaking technology for genome editing is the CRISPR-Cas system which was inspired by the bacterial adaptive immunity against invading bacteriophages. In August 2012, the groups of Jennifer A. Doudna at the University of California, Berkeley, and Emmanuelle Charpentier at the Umea University in Sweden (now at the Max Planck Institute of Infection Biology in Berlin) [10] showed for the first time that a monomeric DNA endonuclease, known as Cas9, from* Streptococcus pyogenes* can be easily programmed to cut double-stranded DNA at a specific genomic sequence using complementary base pairing of a single-guide RNA (sgRNA, Figure 1). The potential to exploit this simple system for genome editing in eukaryotic systems (human, mouse) was demonstrated few months later by the work of Feng Zhang's group at Massachusetts Institute of Technology (MIT) [11] and George Church's group at Harvard University [12]. In these studies, only a single construct for expression of Cas9 nuclease and a specifically designed sgRNA are needed for transformation. Since then, due to its ease of implementation and robustness the CRISPR-Cas9 system has been utilized widely for genome engineering in various organisms, including plants [13–15], insects [16], fish [17], rabbits [18], pigs [19], mice [20], monkeys [21], and human cells [22, 23]. A large number of publications using the CRISPR-Cas9 technology came up rapidly since the first reports and promoted our understanding and applications of the system. Here we review the major advances in plant genome editing technology using Cas9 RNA-guided endonuclease (RGEN) and discuss its applications as well as future prospects in molecular plant breeding.

## 2. Overview of the Genome Editing CRISPR-Cas9 System 

The immense versatility of the CRISPR-Cas9 technology in the field of genome editing is due to its simplicity, efficiency, and robustness. Basically, the CRISPR-Cas9 tool consists of two main components, deliverable as a single plasmid (Figure 1(A)): a bacterial Cas9 endonuclease protein and a specifically designed sgRNA containing a 20-bp sequence homologous to the target DNA (called protospacer). A prerequisite for cleavage of the target DNA is the presence of a sequence 5′-NGG-3′ [10] or 5′-NAG-3′ [24] as the conserved protospacer-adjacent motif (PAM). Importantly, it has been shown that multiple sgRNA targeting to different genomic loci can be simultaneously exploited to achieve high-efficiency multiplex genome engineering without requiring additional Cas9 proteins [11, 12]. Moreover, some initial in vitro and in vivo evidence suggested that Cas9 endonuclease activity is not affected by DNA CpG methylation [24]. However, sgRNA preferentially binds to open chromatin regions including off-target sites [25, 26]. Further efforts to improve our understanding of the effect of chromatin accessibility and epigenetic environment at the target site on the efficiency of the CRISPR-Cas9 system are needed.

The main concern regarding the implementation of the CRISPR-Cas9 system for genome editing is occasional off-target modifications reported in some studies [11, 12, 24, 27–29]. Although a 20 bp recognition sequence in the sgRNA was initially considered necessary to determine specificity, it was later shown that a perfect match between the 7–12 bp at the 3′ end of the sgRNA (called the seed region) and the equivalent region of the target DNA confers target site recognition and cleavage, whereas multiple mismatches in the PAM-distal region are generally tolerated [24, 27–29]. Several strategies have been developed to control the specificity of CRISPR-Cas9, in which the design of the sgRNA is considered as an important and easily implementable one. A number of guidelines and online tools have been developed to facilitate the selection of unique target sites in organisms for which high quality whole genome sequences are available [24, 30, 31]. Truncated sgRNA with length of 17 bp or elongated sgRNA with 2 additional guanidine residues at the 5′ end could reduce nontarget mutations [32, 33]. Low expression level of Cas9 nuclease is another way to reduce off-target activities [29, 34].

The most widely used Cas9 nuclease originates from the type II (class 2) CRISPR-Cas9 system of* Streptococcus pyogenes *(SpCas9). However, Cas9 orthologues from other bacterial species are also applicable and may offer further optimization of the current CRIPSR-Cas9 system. For instance, Cas9 gene of* Staphylococcus aureus* (SaCas9), which is 1 kb shorter than that of* S. pyogenes,* could improve its stability in transformation vectors [35]. Interestingly, SaCas9 targets another distinct PAM 5′NNGGGT3′. On the other hand, a new endonuclease of the class 2 CRISPR-Cas systems, Cpf1 (CRISPR from* Prevotella* and* Francisella* 1), has been reported to require a T-rich PAM motif upstream of the target site and generates a DSB with 5′ overhangs [36]. Finding of further Cas9 nucleases which require different PAM motifs might allow targeting of more diverse genomic positions and enable harnessing more complex applications for genome engineering by using combination of these Cas9.

Detailed understanding of the molecular structure of the CRISPR-Cas9 systems guides us to rationally redesign and customize variants of Cas9 enzymes. Crystal structures of SpCas9, SaCas9,* Francisella novicida* Cas9 (FnCas9), or Cpf1 in complex with their sgRNA and double-stranded target DNAs [37–41] were solved, revealing distinct mechanisms of PAM recognition and of RNA-guided DNA targeting by Cas9 nucleases. Several engineered CRISPR-Cas9 variants were produced to increase Cas9 specificity or to alter the PAM recognition patterns [42]. Cas9 nickase variants (Cas9-D10A or Cas9-H840A) containing a single inactive nuclease domain cleave only one DNA strand to create a single-strand break at the target sites. A pair of induced nicks, one on each strand and up to 100 bp apart from each other, can result in a DSB with overhang. This approach using Cas9 nickase could significantly reduce the off-target mutation rate [43, 44]. In addition, fusion of catalytically inactive Cas9 (Cas9-D10A-H840A, dCas9) and FokI nuclease, which functions only as a dimer, showed comparable results when they are guided by a pair of sgRNAs [45, 46]. Interestingly, dCas9 could be exploited not only in genome editing but also in many other applications, such as modifications of gene expression [47], epigenetic editing [48], and visualization of specific DNA sequences in living cells [49].

## 3. Major Advances of Plant and Crop Genome Editing Technology Using Cas9 RNA-Guided Endonucleases (RGENs)

The CRISPR-Cas9 system with the ability to precisely cut DNA of essentially any organism provides an unprecedented tool for genomic engineering. Soon after the evidence that the CRISPR-Cas9 system works in animal models, three papers reported expression and activities of the plant-codon-optimized CRISPR-Cas9 in plant model species of* Arabidopsis* (*Arabidopsis thaliana*) and tobacco (*Nicotiana benthamiana*) as well as in crops such as rice and wheat [50–52]. Those first groups demonstrated the versatility of the technology by using different transient or stable transformation platforms (protoplast transfection, leaf agroinfiltration, and particle bombardment of callus) in order to generate small deletions and/or insertions, targeted insertions, and multiplex genome modifications. Furthermore, the transmission to progeny and Mendelian heritability of CRISPR-Cas9-induced mutations was shown by using the* Agrobacterium*-mediated germ line transformation in* Arabidopsis* [14, 15, 53, 54] and rice [55–58], suggesting that the CRISPR-Cas9 system could become a powerful tool in crop genome editing. Subsequent work reported successful applications of the CRISPR-Cas9 tool for sorghum [13], wheat [59], maize [60], sweet orange [61], tomato [62], potato [63], liverwort* Marchantia polymorpha* L. [64], barley and* Brassica oleracea* [65], soybean [66], melon [67], and poplar [68]. Summarizing information about transformation/delivery methods and expression systems for CRISPR-Cas9-based applications in plants can be found in recent reviews [69, 70]. It is needed to emphasise that spreading of this technology is highly promoted by the CRISPR research community, providing open access to plasmids, web tools, and active discussion groups (Table 1).

Since plant genomes are large, complex, and often polyploid, off-target mutations can be expected to happen during genome engineering. When introducing a gRNA-Cas9 cleavage in rice, Xie and Yang [71] reported a mutation rate of 1.6% at a single off-target sequence which has a single mismatch at position 15 bp proximal to the PAM. Off-target effects of CRISPR-Cas9 have been observed in other plant species, including soybean [72], maize [73], and barley and* B. oleracea* [65]. In contrast, no off-target mutation events could be detected at the putative off-target sites in studies on* Arabidopsis*, tobacco, wheat, rice, or sweet orange [14, 50–52, 58, 59, 61], even using whole-genome sequencing [14]. Notably, off-target events are less problematic in plant breeding than for clinical research because off-target mutations can be segregated away from the mutation of the target by crossing mutants with wild-type plants. However, the crossing procedure can be laborious, time-consuming, or even impossible for perennial plants and vegetatively propagated crops, such as potatoes, bananas, and cassava. The off-target problem can be overcome either by optimizing sgRNA design [74, 75] or by using high fidelity CRISPR-Cas9 approaches with more precise Cas9 variants (e.g., [76]), paired Cas9 nickases, or dCas9:FokI fusions. The application of the Cas9-D10A nickase in* Arabidopsis* suggests off-target effects might be avoided by using a pair of nickases [15, 77].

The primary application of CRISPR-Cas9 technology in genome editing (or reverse genetics studies) is gene knockout because the host cells preferentially repair the Cas9-induced DSBs via the “error-prone” NHEJ pathway which often results in short insertions or deletions. The size of these modifications and the ratio between insertions and deletions could have an impact on genome size and direct genome size evolution [78, 79]. The flexibility of the CRISPR-Cas9 tool enables targeting of adjacent sites in chromosome for specific removal of a large unwanted DNA sequences, from several kb in* Arabidopsis* [52, 53], tobacco [30], and tomato [62] up to 245 kb in rice [58]. CRISPR-Cas9 can also be utilized to knockout multiple genes of gene family in rice [80] or homoeologous genes in hexaploid bread wheat [81, 82]. A CRISPR-Cas9 toolbox for multiplexed genome editing was demonstrated in model plants such as* Arabidopsis*, tobacco, and rice [83]. In order to perform highly multiplexing genome manipulations, CRISPR-Cas systems using Cas9 orthologues of* Staphylococcus aureus* (SaCas9) and* Streptococcus thermophilus* (St1Cas9) have been adapted for use in* Arabidopsis* [84]. In addition, modified Cas9 variants enable targeting to noncanonical PAM sites in rice [85], providing a wider range of genome editing.

For site-specific gene insertion (“trait stacking”) or replacement it is needed to exploit the HR pathway for repairing the Cas9-induced DSB. HDR events require as template a sequence homologous to the target gene (Figure 1, [8]). However, HDR frequency in CRISPR-Cas9-mediated gene targeting (GT) is rather low as shown in rice [50], in tobacco [52], and in* Arabidopsis* [53, 55]. By using approximately 670 bp homology on either side of the break, Schiml et al. [77] could insert a 1.8 kb marker gene into an endogenous gene of* Arabidopsis* with a frequency of 0.14%. In order to overcome the low HDR efficiency of targeted genome manipulation in mammalian cells, components of the NHEJ pathway were inhibited [86]. Similarly, by manipulating of DNA ligase IV (a member of the NHEJ pathway), CRISPR-Cas9-induced HDR-mediated GT can work more efficiently in rice, resulting in biallelic plants [87]. Alternatively, rational design of orientation, polarity, and length of the donor ssDNA to match the properties of the Cas9-DNA complex could increase the HDR events [88]. Ideally, the efficiency of HDR-mediated genome modifications would be improved by delivery of sufficient quantities of the donor sequence for HDR repair at the Cas9-targeted site. A transformation method using a nuclear replicating DNA virus [89] which produces multiple copies of the donor sequences for HDR inside plant cells has recently been demonstrated to generate high-frequency, precise genome modifications in tomato [90].

## 4. Future Perspectives of the CRISPR-CAS Technology for Plant Breeding

The CRISPR-Cas9 technology is revolutionizing genome engineering and equipping scientists and breeders with the ability to precisely modify the DNA of crop plants. Importantly, CRISPR-Cas9 enables genome modifications also in potential crop plants for which genetic manipulation has been a challenge (e.g., duckweed [91]), provided that high quality whole genome sequences [92, 93] and efficient transformation procedures are available [94]. This review does not cover ethical, legal, and social issues of this revolutionary tool (for such aspects, see [2–4, 95]). In this context, it is necessary to note that the common white button mushroom (*Agaricus bisporus*) that has been modified to resist browning using CRISPR-Cas9 became the first CRISPR-edited organism that can be cultivated and sold without further oversight of US regulations [96]. Interestingly, the CRISPR-Cas9 approach offers genetic manipulation of crops without transgenic footprints by delivering preassembled Cas9-sgRNA ribonucleoproteins [97] or by transient expression of the in vitro transcripts of Cas9-coding sequence and sgRNA [82] and thus might not be classified as genetically modified organisms and regulated by existing biosafety regulations.

A major power of CRISPR/Cas9-induced genome editing is to provide an opportunity for targeting multiple sites simultaneously. New application of this technology is conferring multiple pathogen resistances to crop plants. By establishing CRISPR-Cas9-like immune systems, tobacco and* Arabidopsis* were made resistant to the beet severe curly top virus [98], the bean yellow dwarf virus [99], and the tomato yellow leaf curl virus, respectively [100]. The recently developed CRISPR-Cas system with programmable RNA recognition and cleavage [101] would be exciting to apply in plants because the majority of plant viruses are RNA viruses [102]. However, further studies will be required to monitor the stability of such resistances over generations and in diverse habitats [103].

CRISPR-Cas9 has triggered innovative applications in several fields, including agriculture. CRISPR-edited individual organisms could spread a positively selectable gene throughout a wild population in a so-called gene drive process. In principle, such CRISPR-based gene drive systems could be beneficial to mankind, for example, by potentially preventing the spread of diseases, or supporting agriculture by reversing pesticide and herbicide resistance in insects and weeds, and control damaging invasive species [104]. Gene drives will work only in sexually reproducing species and spread significantly only in species that reproduce quickly. The gene drive model has been tested in yeast [105] and the first CRISPR-Cas9-engineered mosquitoes have recently developed to fight malaria [106]. However, because of low efficient homologous recombination, gene drive application to either eliminate or reduce invasive plant species in a given area is still challenging. In addition, since such technology might pose tremendous alterations to wild populations, biosafety precautions and measures are needed (for more details, see [107, 108]). Importantly, the CRISPR-Cas9-mediated gene drive technology (called as mutagenic chain reaction (MCR) [109]) can be used to produce stable homozygous (biallelic) mutant lines by using HDR-driven propagation of the CRISPR-Cas9-cassette to the companion chromosome. Moreover, such concept can also be applied for editing organelle genomes (e.g., chloroplast) in order to overcome the high copy number of genomes and reversion of mutations [110].

Until now synthetic biology is limited to bacteria models to engineer completely new metabolic pathways. The CRISPR-Cas9 technology opens the way to an easier use of synthetic biology in more complex systems, for example, for agronomical traits in crop plants [111]. Since many complex metabolic pathways in plants interact with each other and are controlled by multiple tissue- or development-specific regulators, metabolic engineering in plants could require not only multiple gene targeting but also probably fine-tuning multiple gene expression level at different tissues or developmental stages. For such sophisticated applications, modifications or customizations of the CRISPR-Cas9 systems including (1) specific Cas9-/sgRNA expression promoters (e.g., [112, 113]), (2) modified Cas9 for alterations of gene expression and epigenetic changes (e.g., [47, 48]), (3) combinations of different Cas9 variants (e.g., [35, 36]) for expanding the target range in the genomes, and (4) efficient technology for increasing HDR-driven precise gene replacement will be needed to be further developed or optimized for particular cell types or organisms. With the rapid development of CRISPR-Cas9 technology during the last 4 years, the promise of a next green revolution with new crops meeting long-standing requests for metabolic engineering (e.g., plants that can fix their own nitrogen, have better nutritious values, can be efficiently utilized for biofuel production, or display enhanced photosynthetic capacity [114]) could be realized in the near future.

## Figures and Tables

**Table 1:** Useful resources/tools for CRISPR-Cas9 research in plants.

Site	Purpose	Authority
http://www.addgene.org	Access to plasmid resource and tutorial documents [115]	Addgene
https://www.protocols.io	Access to detailed protocol resource	Protocols.io
http://cbi.hzau.edu.cn/cgi-bin/CRISPR	Design optimal sgRNA with 43 plant genomes from Ensembl Plants [24]	Huazhong Agricultural University
http://www.genome.arizona.edu/crispr/CRISPRsearch.html	Predict high specific sgRNA of 8 plant genomes [116]	University of Arizona
http://www.rgenome.net/cas-offinder/	Search for potential off-target sites of sgRNA from 37 plant genomes [117]	Institute for Basic Science, Korea
http://www.e-crisp.org/E-CRISP/index.html	Design sgRNA for genome-libraries projects or individual sequences with 11 plant genomes [118]	German Cancer Research Center (DKFZ)
http://crispr.mit.edu/	Find the CRISPR-Cas9 target sites within an input sequence with *Arabidopsis* genome [24]	Zhang Lab, MIT
http://chopchop.cbu.uib.no/	Select CRISPR target sites and predict off-target sites with *Arabidopsis* genome [119]	University of Bergen
http://portals.broadinstitute.org/gpp/public/analysis-tools/sgrna-design	Design highly active sgRNAs for the provided targets [74]	Broad Institute of MIT and Harvard, Cambridge University
http://eendb.zfgenetics.org/casot/	Open-sourced tool for finding potential off-target sites of any user-specific genome [120]	Peking University
https://groups.google.com/forum/#!forum/crispr	Active discussion groups	Google

## Abbreviations

CRISPR: Clustered regularly interspaced short palindromic repeats
Cas9: CRISPR-associated protein 9
DSB: DNA double-strand break
GT: Gene targeting
HR: Homologous recombination
NHEJ: Nonhomologous end-joining
sgRNA: Single-guide RNA
PAM: Protospacer-adjacent motif
RGEN: RNA-guided endonuclease.
